# Effectiveness of Insulin Pump Therapy Versus Multiple Daily Injections for Glycemic Control and Rate of Diabetic Ketoacidosis Among Children With Type 1 Diabetes Mellitus

**DOI:** 10.7759/cureus.54123

**Published:** 2024-02-13

**Authors:** Nusaybah Alnaim, Hussain A Al Ghadeer, Abdulhameed A Al-Bunyan, Abdulmohsen Almulhem, Yassin Alsaleh, Manal AlHelal, Ishaq Almugaizel, Zahra Alhashim, Ahmed M Alhamrani, Zinab A Al Bosrour

**Affiliations:** 1 Endocrinology and Diabetes, Maternity and Children Hospital, Al Ahsa, SAU; 2 Pediatrics, Maternity and Children Hospital, Al Ahsa, SAU; 3 Endocrinology and Diabetes, King Faisal General Hospital, Al Ahsa, SAU

**Keywords:** alahsa, saudi arabia, dka, glycaemic control, multiple daily injections (mdi), continuous subcutaneous insulin infusion (csii), insulin pump, diabetes mellitus

## Abstract

Background

Advances in pump technology and the availability of insulin analogs, as well as the results of the Diabetes Control and Complications Trial (DCCT), which established the benefit of improved glycemic control, have all contributed to the increased use of insulin pump therapy in recent years, particularly in children.

Purpose

This research aims to compare the impact of insulin delivery method, i.e., continuous subcutaneous insulin infusion (CSII) or multiple daily injections (MDI) on glycemic control and the rate of diabetic ketoacidosis (DKA) among children with type 1 diabetes mellitus in Al Ahsa, Saudi Arabia.

Methods

A retrospective cohort study was carried out in a diabetic center in Al Ahsa, Saudi Arabia, over 24 months (2020-2022) among children with type I diabetes mellitus (age group 1-14 years).

Results

In total, 351 patients with diabetes were induced, with 316 (90%) on MDI and 35 (10%) on CSII. After six months of diagnosis, precisely 38 (12%) of patients with diabetes on the MDI regimen experienced DKA, compared to 4 (11.4%) of those on the CSII regimen, with no statistically significant difference (P=0.918). At six months and nine months of follow-up, the average hemoglobin A1c (HbA1c) was considerably higher in diabetic patients on MDI (8.9 ± 1.7% vs. 8.2 ± 1.5% and 9.1 ± 1.6% vs. 8.0 ± 1.3%, respectively, with a significant p-value ≤0.05).

Conclusion

In this study, we found that patients on the MDI regimen had considerably higher HbA1c levels than patients on the CSII regimen, but there was no statistically significant difference in DKA rates between them. This is a short-term follow-up study, and we recommend that patients be followed for a longer period of time for further accurate outcomes.

## Introduction

Type 1 diabetes mellitus (T1DM) is a chronic autoimmune illness that is characterized by an inability to produce insulin due to an autoimmune destruction of beta cells in the pancreas [1]. Diabetes mellitus treatment can result in social and financial burden for the family and the health system [2]. Type 1 diabetes has a number of documented macrovascular and microvascular complications; it is considered that achieving adequate glycemic control is one of the strategies to minimize the occurrence of complications [3,4]. Patients on the continuous subcutaneous insulin infusion (CSII) regimen, particularly those on the continuous glucose monitoring sensor (CGMS), are believed to have superior control over their glycemic monitoring and the frequency of diabetic ketoacidosis (DKA) events as compared to patients on the multiple daily injections (MDI) regimen, despite having distinct types of insulin infusion [5-7].

The main principles of management of type I diabetes mellitus are monitoring blood glucose levels (BGLs) and administering intensive insulin therapy to achieve optimal glycemic control. By modifying the insulin dosage in response to blood glucose readings, patients with type 1 diabetes can achieve better glycemic control with continued glucose monitoring. The finger glucocheck method of blood glucose monitoring presented challenges for the patient's family because of the social application and pain. But because continuous glucose monitoring (CGM) has an alarm system for hypo- and hyper-glycemia, it not only helps the patient with their pain but also makes blood glucose monitoring easier for the family [8,9]. Although there are several ways to make insulin administration easier for families, such as insulin ports and jets, the majority of clinical centers still treat patients with multiple daily injections (MDI) of insulin analogs as the standard of care [10]. The insulin pump is a unique therapeutic approach for type 1 diabetes that has seen significant advancements over the years. Some studies demonstrate that CSII is superior to MDI in decreasing hemoglobin A1c (HbA1c), while others show no significant difference [11,12]. Pediatric diabetic patients had numerous challenges in managing blood glucose [13], which may be attributed to patient age, increased insulin requirements in relation to developing children, and social factors that may influence teenager complaints.

The purpose of this study was to assess the efficacy of CSII versus MDI in children with type I diabetes mellitus at our diabetes center in Al Ahsa City, utilizing glycemic control and DKA incidents as outcome measures.

## Materials and methods

Objectives

The primary goal of this study was to compare insulin pump therapy, commonly known as CSII, with MDI in terms of effectiveness in managing T1DM in pediatric patients as measured by HbA1c levels and DKA rate at the diabeter center in Al Ahsa, Saudi Arabia.

Study area

The diabetic center at Al Ahsa, also known as the diabeter, is a certified facility that meets international standards and is committed to offering personalized, comprehensive care to children and young adults with type 1 diabetes. Our center is recognized as one of the largest diabetes-specialized centers in Saudi Arabia and the Netherlands. Our facility is currently taking care of hundreds of patients. In 2020, the center launched, and patient enrollment has begun ever since.

Participants

We obtained the medical records of all children aged 1-14 years who were diagnosed with type 1 diabetes and were treated with CSII or MDI. By the time of analysis, every patient included in this study had been on MDI or CSII for at least nine months, and none of them were in the honeymoon phase following their diagnosis. To prevent additional confounders that could affect glycemic control and the study's outcome, children with diagnoses of other autoimmune diseases, type 2 diabetes, hemoglobinopathy, or bedridden status were excluded.

Study design

In this retrospective cohort study, two groups of young children with T1D who are being followed up at our institute are compared between MDI and CSII. The primary study outcome was glycemic control, which was measured using the HbA1c test every three months for nine months, along with a DKA event. A standardized ion-exchange high-performance liquid chromatography (HPLC) method is used to measure HbA1c. All results are obtained in our center using the same laboratory technician. The patient file's DKA event history was obtained retroactively; it does not include DKA that occurred at the time of diagnosis.

Data analysis

After the data were extracted, it was revised, coded, and fed to the Statistical Software IBM SPSS Version 26 (SPSS, Inc., Chicago, IL). All statistical analysis was done using two-tailed tests. A P value less than 0.05 was statistically significant. Descriptive statistics using mean with standard deviation was for scale variables and continuous variables, such as age, HbA1c, and BMI, while frequency and percent were used for categorical variables, such as gender and mode of insulin delivery (MDI vs. CSII). An independent t-test was used to compare HbA1c levels between the two groups (MDI vs. CSII). The chi-square test and an exact test were used to compare categorical variables if the sample size was small.

## Results

A total of 351 diabetic patients were induced; 316 (90%) were on MDI and 35 (10%) were on CSII (Table 1). The mean age was 9.8 ± 2.7 years for the MDI group compared to 11.1 ± 2.0 years for the CSII group, with no statistical difference (P=0.069). Approximately 168 (53.2%) of the patients with diabetes on MDI were females versus 25 (71.4%) of others on CSII with recorded statistical significance (P=0.039). Also, BMI was significantly higher among CSII patients in comparison to the other patients on MDI (19.3 ± 3.9 vs. 17.7 ± 3.7, respectively; P=0.016). 

**Table 1:** Bio-demographic data of study diabetic patients’ according to their management regimen. P: Pearson X^2^ test; $: Exact probability test; #: Independent t-test; *P<0.05 (significant).

Personal data	Regimen				p-value
	MDI		CSII		
	No	%	No	%	
Age in years					0.069
<6	43	13.6%	2	5.7%	
7-10	120	38.0%	9	25.7%	
11-15	153	48.4%	24	68.6%	
Mean ± SD	9.8 ± 2.7		11.1 ± 2.0		
Gender					0.039*
Male	148	46.8%	10	28.6%	
Female	168	53.2%	25	71.4%	
Hemoglobinopathies and chronic diseases					0.102^$^
None	278	88.0%	34	97.1%	
Hyperlipidemia	38	12.0%	1	2.9%	
BMI					0.016*^#^
Mean ± SD	17.7 ± 3.7		19.3 ± 3.9		

DKA among diabetic patients on the CSII versus MD regimen (Table 2). An exact 38 (12%) of diabetic patients on the MDI regimen had DKA after six months of diagnosis compared to 4 (11.4%) of others on the CSII regimen with no statistical significance (P=0.918). Regarding causes of DKA, the missed dose was reported among 11 (45.8%) MDI regimen patients compared to none of the CSII regimen patients. Also, 1 (33.3%) of patients on the MDI regimen reported uncontrolled BGL compared to none of those on the CSII regimen. Infection was reported as a cause of DKA among 66.7% of diabetic patients on CSII compared to 1 (4.2%) of those on MDI, while 1 (33.3%) of patients on CSII reported for other causes with recorded statistical significance (P=0.007).

**Table 2:** The rate of DKA among diabetic patients on CSII versus MDI regimen. P: Exact probability test; *P<0.05 (significant); DKA: diabetic ketoacidosis; CSII: continuous subcutaneous insulin infusion; MDI: multiple daily injections.

Diabetic ketoacidosis	Regimen				p-value
	MDI		CSII		
	No	%	No	%	
DKA after six months from the diagnosis					0.918
Yes	38	12.0%	4	11.4%	
No	278	88.0%	31	88.6%	
Causes of DKA					0.007*
Missed dose	11	45.8%	0	0.0%	
Uncontrolled blood glucose levels	8	33.3%	0	0.0%	
Infection	1	4.2%	2	66.7%	
Others	4	16.7%	1	33.3%	

**Figure 1:**
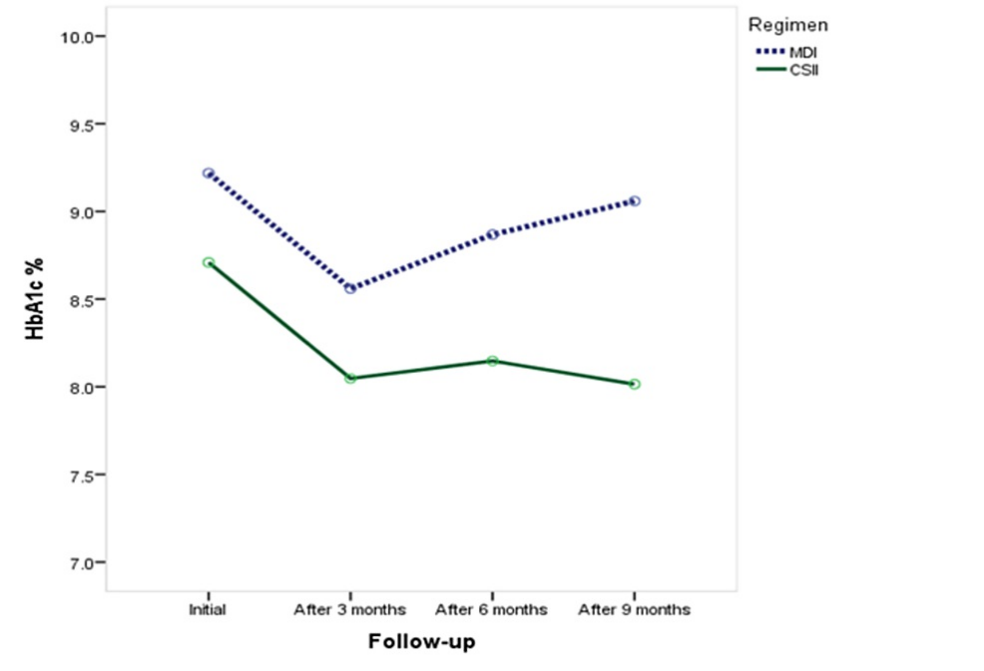
HbA1c among diabetic patients on CSII versus MDI regimen. HbA1c: hemoglobin A1c; MDI: multiple daily injections; CSII: continuous subcutaneous insulin infusion.

HbA1c among diabetic patients on the CSII versus MDI regimen (Table 3, Figure 1). The average HbA1c was significantly higher among patients on the MDI regimen measured outside the diabetic center (9.6 ± 2.2% vs. 8.8 ± 2.5%, respectively; P=0.045). Also, it was significantly higher among diabetic patients on the MDI regimen at six months and nine months of follow-up (8.9 ± 1.7% vs. 8.2 ± 1.5% and 9.1 ± 1.6% vs. 8.0 ± 1.3%, respectively).

**Table 3:** HbA1c among diabetic patients on CSII versus MDI regimen. P: Independent samples t-test; *P<0.05 (significant); HbA1c: hemoglobin A1c; CSII: continuous subcutaneous insulin infusion; MDI: multiple daily injections.

Hemoglobin A1c (HbA1c)	Regimen				p-value
	MDI		CSII		
	Mean	SD	Mean	SD	
HbA1c outside the diabetic center	9.6	2.2	8.8	2.5	0.045*
HbA1c inside the diabetic center	9.2	1.9	8.7	1.8	0.142
HbA1c after three months of follow-up in the diabetic center	8.6	1.8	8.0	1.6	0.107
HbA1c after six months of follow-up in the diabetic center	8.9	1.7	8.2	1.5	0.021*
HbA1c after nine months of follow-up in the diabetic center	9.1	1.6	8.0	1.3	0.001*

HbA1c among diabetic patients on the CSII versus MD regimen by their gender (Table 4). Among male diabetic patients, there was no significant difference regarding HbA1c at any follow-up phase. In contrast, HbA1c was significantly higher among female diabetic patients on the MDI regimen than others on the CSII regimen at six months and nine months of follow-up.

**Table 4:** HbA1c among diabetic patients on CSII versus MD regimen by their gender. P: Independent samples t-test; *P<0.05 (significant); HbA1c: hemoglobin A1c; MDI: multiple daily injections; CSII: continuous subcutaneous insulin infusion.

HbA1c	Male				p-value	Female				p-value
	MDI		CSII			MDI		CSII		
	Mean	SD	Mean	SD		Mean	SD	Mean	SD	
HbA1c outside the diabetic center	9.7	1.8	8.9	1.7	0.148	9.5	2.5	8.8	2.8	0.189
HbA1c inside the diabetic center	9.3	1.8	8.7	2.0	0.340	9.2	2.0	8.7	1.7	0.285
HbA1c after three months of follow-up in the diabetic center	8.5	1.8	8.3	1.9	0.744	8.6	1.8	7.9	1.5	0.084
HbA1c after six months of follow-up in the diabetic center	9.0	1.7	8.5	1.6	0.379	8.8	1.6	8.1	1.5	0.041*
HbA1c after nine months of follow-up in the diabetic center	9.1	1.6	8.3	1.2	0.120	9.0	1.6	7.9	1.3	0.001*

## Discussion

The current study aimed to evaluate the effectiveness of insulin pump use in the pediatric age group in comparison to the MDI regimen and whether to decrease or increase the rate of DKAs. According to recent studies, there is ongoing debate about the effectiveness of insulin pump therapy versus multiple daily injections (MDIs) in the management of type 1 diabetes mellitus. Insulin pump therapy has been found to be effective in controlling blood sugar levels in people with diabetes [14]. In fact, it has been shown to provide better glycemic control and reduce the incidence of hypoglycemia compared to traditional multiple daily injections [15,16]. Insulin pumps offer a continuous supply of insulin, which more closely simulates the body's natural insulin response to food and helps to reduce blood sugar variability. However, insulin pumps may not be suitable for everyone and can come with a steep learning curve [17]. It is important to work with a healthcare professional to determine if an insulin pump is the right choice for managing diabetes. With regard to DKA, the study results showed that the incidence was nearly equal among the two groups of diabetic patients with no statistical significance. The only causes of DKA showed a significant difference between the two groups, where the missed dose was reported among less than half of MDI regimen patients compared to none of the CSII regimen patients. Also, one-third of patients on the MDI regimen reported uncontrolled BGL compared to none of those on the CSII regimen. Infection was reported as a cause of DKA among two-thirds of diabetic patients on CSII compared to very few cases on MDI, while one-third of patients on CSII reported other causes with recorded statistical significance. This indicates that the daily regimen was associated with missing doses, which resulted in uncontrolled blood glucose levels, while insulin pumps may be associated with different factors rather than the need for frequent injections. Regarding diabetic control, the study results showed that there was a statistically but not clinically significant difference regarding HbA1c among the two groups, where both showed similar measures within six and nine months of follow-up with some advantage for insulin pump therapy. Similar findings were reported by Garg et al. [18], where patients with type 1 diabetes can achieve similar glycemic control using insulin glargine with premeal insulin lispro or by using an external infusion pump with insulin lispro or insulin apart. However, costs and episodes of diabetic ketoacidosis are significantly higher for insulin pump users. Also, Raskin et al. [19] also failed to report a clear advantage to CSII treatment in a large, multicenter, open-label, randomized study including obese and uncontrolled type 2 diabetic patients. Similarly, Yardley et al. [20] found that both MDI and CSII groups had similar reductions in glucose levels during exercise, but responses in early and late recovery differed. Participants using MDI had greater increases in glucose throughout recovery compared with individuals with CSII. Two-thirds of the MDI patients experienced late-onset post-exercise hyperglycemia (blood glucose >12 mmol/L) compared with only 1/10th of the CSII patients (P<0.01). Additionally, the REPOSE Study Group [21] showed that both groups showed clinically relevant and long-lasting decreases in HbA1c, rates of severe hypoglycemia, and improved psychological measures.

In Saudi Arabia, Riyadh, Babiker et al. [5] conducted a study and found that the CSII group consistently had lower HbA1c levels compared to the MDI group throughout a three-year follow-up period: 8.1% versus 10.1 at one year, 7.5% versus 10.1% at two years, and 8.9% versus 10.3% at three years. Other studies that compared CSII to MDI revealed that most of the improvement in HbA1c was recorded in the first few months after the initiation of CSII [22-24]. Steineck et al. [25] documented that the risk of cardiovascular disease was significantly lower among patients on a multiple daily injection regimen compared to insulin pump treatment.

This study has several limitations. Because this was a nonrandomized, observational study, it was susceptible to selection bias. The level of diabetes education, motivation, family support, and mental health issues were not taken into consideration. Additionally, the small number of patients on CSII. As a result, generalizing the findings of this single-center study was limited. Therefore, we highly recommend that future studies look for more conclusive evidence on the efficacy of MDI vs. CSII by enrolling several centers in Saudi Arabia.

## Conclusions

In our patients, the use of CSII may be linked with better glycemic control. These data may prove helpful in future research and/or clinical practice, particularly in light of the rapid advancements in diabetes technology, including the development of autonomous artificial pancreas systems. Our data support the long-term benefit of CSII on glycemic control in real-life uncontrolled settings, which may translate to lower rates of diabetes complications.

## References

[1] Paschou SA, Papadopoulou-Marketou N, Chrousos GP, Kanaka-Gantenbein C (2018). On type 1 diabetes mellitus pathogenesis. Endocr Connect.

[2] Berezin A (2016). Metabolic memory phenomenon in diabetes mellitus: achieving and perspectives. Diabetes Metab Syndr.

[3] White NH, Sun W, Cleary PA (2008). Prolonged effect of intensive therapy on the risk of retinopathy complications in patients with type 1 diabetes mellitus: 10 years after the Diabetes Control and Complications Trial. Arch Ophthalmol.

[4] Nimri R, Weintrob N, Benzaquen H, Ofan R, Fayman G, Phillip M (2006). Insulin pump therapy in youth with type 1 diabetes: a retrospective paired study. Pediatrics.

[5] Babiker A, Alammari N, Aljuraisi A, Alharbi R, Alqarni H, Masuadi E, Alfaraidi H (2022). The effectiveness of insulin pump therapy versus multiple daily injections in children with type 1 diabetes mellitus in a specialized center in Riyadh. Clin Med Insights Endocrinol Diabetes.

[6] Benkhadra K, Alahdab F, Tamhane SU, McCoy RG, Prokop LJ, Murad MH (2017). Continuous subcutaneous insulin infusion versus multiple daily injections in individuals with type 1 diabetes: a systematic review and meta-analysis. Endocrine.

[7] Hanas R, Lindgren F, Lindblad B (2009). A 2-yr national population study of pediatric ketoacidosis in Sweden: predisposing conditions and insulin pump use. Pediatr Diabetes.

[8] Khan AM, Alswat KA (2019). Benefits of using the i-Port system on insulin-treated patients. Diabetes Spectrum.

[9] Engwerda EE, Tack CJ, de Galan BE (2013). Needle-free jet injection of rapid-acting insulin improves early postprandial glucose control in patients with diabetes. Diabetes Care.

[10] Pala L, Dicembrini I, Mannucci E (2019). Continuous subcutaneous insulin infusion vs modern multiple injection regimens in type 1 diabetes: an updated meta-analysis of randomized clinical trials. Acta Diabetol.

[11] Plotnick LP, Clark LM, Brancati FL, Erlinger T (2003). Safety and effectiveness of insulin pump therapy in children and adolescents with type 1 diabetes. Diabetes Care.

[12] Karges B, Schwandt A, Heidtmann B (2017). Association of insulin pump therapy vs insulin injection therapy with severe hypoglycemia, ketoacidosis, and glycemic control among children, adolescents, and young adults with type 1 diabetes. Jama.

[13] Hill-Briggs F, Adler NE, Berkowitz SA (2020). Social determinants of health and diabetes: a scientific review. Diabetes Care.

[14] Berget C, Messer LH, Forlenza GP (2019). A clinical overview of insulin pump therapy for the management of diabetes: past, present, and future of intensive therapy. Diabetes Spectrum.

[15] Bergenstal RM, Tamborlane WV, Ahmann A (2010). Effectiveness of sensor-augmented insulin-pump therapy in type 1 diabetes. N Engl J Med.

[16] Slover RH, Welsh JB, Criego A, Weinzimer SA, Willi SM, Wood MA, Tamborlane WV (2012). Effectiveness of sensor-augmented pump therapy in children and adolescents with type 1 diabetes in the STAR 3 study. Pediatr Diabetes.

[17] Pickup JC (2012). Insulin-pump therapy for type 1 diabetes mellitus. N Engl J Med.

[18] Garg SK, Walker AJ, Hoff HK, D'Souza AO, Gottlieb PA, Chase HP (2004). Glycemic parameters with multiple daily injections using insulin glargine versus insulin pump. Diabetes Technol Ther.

[19] Raskin P, Holcombe JH, Tamborlane WV (2001). A comparison of insulin lispro and buffered regular human insulin administered via continuous subcutaneous insulin infusion pump. J Diabetes Complications.

[20] Yardley JE, Iscoe KE, Sigal RJ, Kenny GP, Perkins BA, Riddell MC (2013). Insulin pump therapy is associated with less post-exercise hyperglycemia than multiple daily injections: an observational study of physically active type 1 diabetes patients. Diabetes Technol Ther.

[21] REPOSE Study Group (2017). Relative effectiveness of insulin pump treatment over multiple daily injections and structured education during flexible intensive insulin treatment for type 1 diabetes: cluster randomised trial (REPOSE). BMJ.

[22] Nabhan ZM, Kreher NC, Greene DM, Eugster EA, Kronenberger W, DiMeglio LA (2009). A randomized prospective study of insulin pump vs. insulin injection therapy in very young children with type 1 diabetes: 12-month glycemic, BMI, and neurocognitive outcomes. Pediatr Diabetes.

[23] Korkmaz Ö, Demir G, Çetin H (2018). Effectiveness of continuous subcutaneous insulin infusion pump therapy during five years of treatment on metabolic control in children and adolescents with type 1 diabetes mellitus. J Clin Res Pediatr Endocrinol.

[24] Jakisch BI, Wagner VM, Heidtmann B (2008). Comparison of continuous subcutaneous insulin infusion (CSII) and multiple daily injections (MDI) in paediatric type 1 diabetes: a multicentre matched-pair cohort analysis over 3 years. Diabet Med.

[25] Steineck I, Cederholm J, Eliasson B (2015). Insulin pump therapy, multiple daily injections, and cardiovascular mortality in 18,168 people with type 1 diabetes: observational study. BMJ.

